# Coition in Pregnancy

**Published:** 1881-08

**Authors:** Theophilus Parvin


### Selections.

### Coition in Pregnancy.

By Theophilus Parvin, M. D.

Popilia, when reminded that pregnant animals did not permit the approaches of the male, frankly replied, "It is because they are brutes."

Undoubtedly abstinence from coition, once the design of this function has been accomplished, is the law of nature. Ought the human race to accept this law as governing its action?

Recent obstetric writers are generally silent upon the question; occasionally some half-breed—borrowing a term from Albany—writing medicine for the mass, sustains the negative, often qualifying the permission to indulge with certain cautions; but upon the whole there seems a tacit consent for the laity to settle the question as, guided by wise reason and kind sympathy on the one hand or by blind instinct and ungoverned passion on the other, they choose, just as my good friend the late Dr. M. B. Wright once said to me, "We must leave these matters to regulate themselves."

Yet our great master Hippocrates thought that pregnant women who abstained from coition had easier labors; Galen dwelt upon the liability to abortion from this cause at certain periods of pregnancy, the fruit more easily detached when more tender and when approaching maturity, so that the Christian Fathers had good authority for their injunction of continence in the early part and toward the end of pregnancy.

The older obstetricians of modern times did not think the matter unworthy of or improper for their consideration. Thus Mauriceau forbade intercourse in the first few days following conception and in the last two months of pregnancy. Dionis, the frank, honest fellow, criticised his reasons and condemned his rules, concluding in these words: "I shall add that Mauriceau made his observations from himself, for though married forty-six years, he did not have a single child. For my part, I have a wife who has been pregnant twenty times and has given me twenty children born favorably at term, and I am persuaded the caresses of the husband do no harm." Gardien, whose contribution to obstetric literature is one of the most valuable and interesting of the century, devotes considerable space to the subject, and in the course of his remarks says: "It probably would be more prudent to abstain from using the rights of marriage from the time that pregnancy is certain up to the end of the lying-in."

The fact that abstinence from sexual congress in pregnancy is the common rule of animals is certainly a strong argument in favor of urging similar abstinence on the part of men. In addition it may be truthfully asserted that the pregnant woman has as little desire for coition as pregnant females of lower orders; nay, oftentimes utterly abhors while submitting, for she is less protected by power of escape.

Furthermore, practitioners are sometimes told by innocent husbands—more rarely by wives who so often suffer in silence—that intercourse causes the latter great pain.

Finally, this is a frequent cause of abortion; at least one-half of the cases of what is termed spontaneous abortion probably are thus produced. Summing up the arguments* in the affirmative of the question, it may be stated that coition in pregnancy is unnatural; so far as woman is concerned, it is generally odious, often painful; and in regard to the newly-created being, frequently murderous.

What can be alleged on the other side? The peace of families and the chastity of husbands are secured by the indulgence. But suppose men were trained to believe that such indulgence is wrong, injurious to others and to themselves, would their amiability and chastity require to be purchased by a momentary pleasure? Would they not rather learn to subdue and rule this otherwise imperious passion? If Newton, Kant, Fontanelle, and Beethoven could live their many honored years with no indulgence of sexual passion, surely other men might abstain a few months without injury!

This ungoverned passion of man is prolific of evil, and, like producing like, the father who never has learned self-control may give his son not only form and feature, but the germ of the same fierce, clamorous desire, which in its full development will prove a heritage of woe to that son and others. That which polite language veils under the designation *social evil*, and which desolates so many happy homes and brings its quick, black harvest of misery, remorse, disease and death, chiefly lives because man does not know aright, does not duly reverence and honor woman, and keep in subjection that which may become one of the master-passions in his heart, and is thus continued from generation to generation.

Surely prospective motherhood, woman within whom proceeds the evolution of the marvelous mysteries of creation,

*It is highly probable that in many instances both the leucorrhea and nausea and vomiting of the early months of pregnancy are greatly increased by coition. Cases have been observed where the nausea and vomiting did not occur at all, or only in a slight degree, if the husband was absent during the pregnancy; while in other pregnancies, he being at home, these symptoms were most distressing.

should be reverenced, is worthy of all kind and thoughtful consideration, and ought to have thrown around her all protective care. The woman who has conceived is *enceinte;* that is, ungirdled—in allusion to the ancient custom of laying aside the girdle when pregnant and placing it in the temple of the gods—at once a preparation for the enlargement of the abdomen and a seeking divine protection. Let her not fail of all human care while in this condition. Nature then offers unto man invitation and opportunity to subordinate passion to reason, to conscience, to will, to a higher love, and thus raise himself above himself. A sensual age claims for coition facilitating parturition; and the most sensual of husbands, finding their wives pregnant very much against their wishes and in spite of the devices of conjugal onanism, will claim that they can now indulge freely and without fear, for matters can be no worse.

We do believe that intercourse in pregnancy has nothing to commend, nothing to excuse itself unto wise men, and that virtuous abstinence on the part of the husband will be a blessing both to him and to his wife and to their posterity.

It may be objected that the abstinence here advocated contradicts almost universal practice—a practice that frequently brings no evil. But how do we know it has no injurious results? Admitting that the wife may, in the majority of cases, not patently suffer—have no miscarriage, no pain, no nausea and vomiting increased or excited thereby—is there no violence done to the finer elements of a refined womanly nature? Does such a woman cheerfully accept it as the way of all, like Hiero's wife, who never perceived her husband's offensive breath, imagining that it was common to all men? It seems that there might follow some lessening of mutual love, respect, reverence.

So far as the husband is concerned, he learns no lessons of self-control, attains no self-mastery in this regard, and mars that ideal manhood which in better hours and with nobler aspirations he seeks to attain. He will be quite ready in such hours to adopt, as applicable to the act, the concluding clause, while he may reject the first, of the following extract from Sir Thomas Brown's *Religio Medici:* "I could be content that we might procreate like trees, without conjunction, or that there were any way to perpetuate the world without this trivial and vulgar way of coition. It is the foolishest act a wise man commits in all his life, nor is there anything that will more deject his cooled imagination when he shall consider what an unworthy piece of folly he hath committed."

As to the other objection, no matter how universal a practice is, if it be wrong, at least endeavor to point out the wrong. Whether I judge from observation, from the great doctrine of evolution which so fascinates the age, or from the power of divinely-revealed truth, the conclusion always is that the world grows better, and that a wiser, higher, happier, nobler generation will one day possess the earth. Each evil pointed out, each wrong discovered, helps the progress to that day, although it may be long before the evil and the wrong cease. Meantime it is a great mistake to accept a popular vote as the criterion of wisdom and right.

Possibly physicians are too reticent in regard to sexual relations, do not consider as fully as they ought the connection of these with human health and happiness, and give that instruction to the people which is so much needed in regard to such relations. Believing this, I can say in the words of Montaigne, "I know very well that few will quarrel with the license of my writings who have not more to quarrel with in the license of their own thoughts."

This may be the voice of one crying in the wilderness, but even in the wilderness many heard. If only truth be uttered, it one day will be heard and heeded by some, and when heard and heeded, will multiply itself a thousand-fold.—*American Practitioner.*

